# Association between Bone marrow lesions & synovitis and symptoms in symptomatic knee osteoarthritis

**DOI:** 10.1016/j.joca.2019.12.002

**Published:** 2019-12-23

**Authors:** T.A. Perry, M.J. Parkes, R.J. Hodgson, D.T. Felson, N.K. Arden, T.W. O’Neill

**Affiliations:** † Centre for Epidemiology Versus Arthritis, Faculty of Biology, Medicine and Health, Manchester Academic Health Science Centre, The University of Manchester, Manchester, UK; ‡ NIHR Manchester Biomedical Research Centre, Manchester University NHS Foundation Trust, Manchester Academic Health Science Centre, Manchester, UK; § Centre for Imaging Sciences, Institute of Population Health, University of Manchester, Manchester, UK; ǁ Department of Rheumatology, Salford Royal NHS Foundation Trust, Salford, UK; ¶ Clinical Epidemiology Research and Training Unit Boston University School of Medicine, Boston, MA, USA; # Nuffield Department of Orthopaedics, Rheumatology and Musculoskeletal Sciences, University of Oxford, Oxford, UK; †† MRC Lifecourse Epidemiology Unit, Southampton University, Southampton, UK

**Keywords:** Osteoarthritis, Symptoms, Bone marrow lesions, Synovitis

## Abstract

**Objective:**

Bone marrow lesions (BMLs) on MRI are typically subchondral in location, however, a proportion occur at knee ligament attachments and also include a cyst-like component. Our aim was to determine whether the volume of BML subtypes and synovial tissue volume (STV) was associated with symptoms in symptomatic knee OA.

**Method:**

Images were acquired in a sub-sample who had taken part in a randomised trial of vitamin D therapy in knee OA (UK-VIDEO). Contrast-enhanced (CE) MRI was performed annually. In those who had ≥1 follow-up and a baseline scan (*N* = 50), STV and BML volume was assessed. BMLs were categorised by location and by the presence/absence of a cyst-like component. WOMAC was assessed annually. We used fixed-effects panel-regression modelling to examine the association between volume and symptoms.

**Results:**

There was no association between knee pain and total subchondral BML volume (*b* = 0.3 WOMAC units, 95% CI −0.3 to 1.0) or total ligament-based BML volume (*b* = 1.9, 95% CI −1.6 to 5.3). The volume of subchondral BMLs with a cyst-like component was not associated with pain (*b* = 0.8, 95% CI −0.5 to 2.1) however, the volume of the cyst-like component itself was associated with pain (*b* = 51.8, 95% CI 14.2 to 89.3). STV was associated with pain (*b* = 2.2, 95% CI 0.6 to 3.7).

**Conclusion:**

The volume of the cyst-like component from subchondral BMLs with a cyst-like component was associated with knee pain. BML location, however, did not influence symptoms. STV was also associated with knee symptoms.

## Introduction

Bone marrow lesions (BMLs), regions of ill-defined hyperintense signal^1,2^, typically occur within the subchondral bone in knee osteoarthritis (OA). BMLs also occur at the central region of the knee and are related to the cruciate ligaments^3^. Such ligamentous (or ligament-based) BMLs are found in up to three quarters of those with symptomatic knee OA and are thought to be possible related to soft tissue injury and trauma^4^. Evidence from observational studies suggest that the presence of BMLs is linked with knee pain, however, to our knowledge there are no data looking at whether ligamentous BMLs are linked with symptoms in knee OA.

A proportion of BMLs include internal regions of well-defined, rounded hyperintense signal on fluid sensitive non contrast-enhanced MRI^5,6^. In histologic study, these regions on magnetic resonance imaging (MRI) have been associated with abnormal joint tissue including mucinous material^2^ and necrotic bone fragments within a surrounding layer of fibrous connective tissue^7^. Due to a lack of evidence supporting the presence of an epithelial component^7,8^, these well-defined, rounded hyperintense signals identified on MRI are commonly referred to as ‘cyst-like/cystic lesions’^2,5,6^ though in other studies are referred to as ‘subchondral/subarticular cysts’^9–11^.

In a small, retrospective study of 32 subjects with repeat MRI data 92% of cystic lesions developed at pre-existing sites of BMLs^6^. In a longitudinal study using data from MOST, BMLs were associated with an increased risk of incident concomitant subchondral cyst-like lesions (OR: 12.9, 95% confidence intervals (CI) 8.9 to 18.6)^5^. In a cross-sectional study using a different sample of the MOST data, regions thought to correspond to subchondral cysts showed evidence of enhancement on contrast-enhanced (CE) MRI with 94.2% of cases showing full enhancement^9^. Data from cross-sectional studies which have assessed such regions semi-quantitatively using methods such as WORMS^11^ suggest no significant association with pain though in one study of 205 subjects the risk was increased though just fell short of statistical significance^12,13^.

To our knowledge there are no data looking at the association between the volume of BMLs with cyst-like components or the volume of the cyst like component itself, assessed quantitatively, and symptoms.

Using data from a recent clinical trial of vitamin D therapy in knee OA (UK VIDEO), the aim of this study was to examine the relationship between the volume of bone marrow lesion (BML) subtypes and symptoms using CE MRI in men and women with symptomatic knee OA. We looked also at the association between synovial tissue volume (STV) and knee symptoms.

## Methods

### Subjects

This was a secondary analysis of a randomised trial. Subjects included in the analysis were participants of the UK VIDEO trial (ISRCTN94818153); a 3-year multicentre, randomised, double-blind, placebo-controlled trial which examined the effect of vitamin D therapy (800 IU/daily) on radiographic joint space narrowing (JSN) in men and women with symptomatic knee OA^14^. Participants were eligible for the trial if over the age of 50 years, with a Kellgren and Lawrence^15^ score of 2–3 and with knee pain for most days in the previous month. Participants were randomised to either oral vitamin D (800 IU/daily) or matching placebo, by study site, using computer-generated number allocation in a 1:1 ratio.

During the trial, a subsample of subjects at one centre (Southampton) had MRIs acquired at baseline and then annually until the end of the study. 50 participants were included in the current analysis - comprising those subjects randomised to both vitamin D and placebo who had; i) a baseline sagittal T_1_-weighted (T_1_-w) fat suppressed (FS) CE MRI and at least one other CE scan over the first 2 years of the study. A further inclusion criterion was that either axial proton-density-weighted (PD-w) FS and/or coronal short tau inversion recovery (STIR) sequences were available for the corresponding visits. In cases of bilateral knee OA, the knee with the smallest joint space width was used.

### Assessments

At baseline and at annual follow-ups, pain, function and stiffness symptoms were assessed using the Western Ontario and McMaster Universities Osteoarthritis Index (WOMAC) questionnaire. Each item was scored on a 100 mm visual analogue scale (VAS) with scores scaled in a negative direction (0 = no pain/disability, 100 = worst pain/disability). WOMAC total was equal to the sum of pain, function and stiffness scores for the given visit. Patients were asked to relate their scores to symptoms experienced within the last 48 hrs.

### Magnetic resonance imaging (MRI)

MRI of the index knee was performed using a 1.5T scanner (Signa (GE Healthcare)) with a phased-array knee coil^16^. The following pulse sequence protocol was used: sagittal T_1_-w FS CE (TR = 600–800 ms, TE = 12.5–16.2 ms, acquisition matrix 256 × 160, slice gap = 0.6 mm, slice thickness = 3 mm), axial proton density FS (TR = 3800–4820 ms, TE = 31.2–32.5 ms, matrix 256 × 192, slice gap = 0.2 mm, slice thickness = 4 mm) and coronal STIR (TR = 3000–4760 ms, TE = 46.1–56.9 ms, matrix 256 × 192, slice gap = 0.3 mm, slice thickness = 3 mm) sequences. Post-contrast sequences were acquired starting 3 min after intravenous injection of gadodiamide (0.2 mL/kg body weight (Omniscan, GE Healthcare)) with all CE scans acquired within 11 min^16^.

### Quantitative assessment

A single reader (TP) blinded to knee pain status performed segmentation of the baseline and follow-up images. The primary sequence used for image segmentation was the sagittal T_1_-w FS CE scan though segmentation was guided by axial and/or coronal sequences. Patient identifiers (i.e., patient ID) were randomised with visits randomised within patients; all randomisation was performed by an external member of the research team who took no part in the evaluation of images or statistical analyses. All visits for a given patient were assessed before moving onto the next subject. Segmentation of the images was performed on a Dell desktop computer (Intel Core 2 Duo, Dell Inc., Round Rock, TX). Intra- and inter-observer agreement was excellent for both BMLs (intra- [3,1] = 0.99 and inter- [3,1] = 0.99) and STV (intra- [3,1] = 0.83 and inter- [3,1] = 0.99)^17^.

#### i) Bone Marrow Lesion (BML) Volume and Sub-types

**Fig. 1:**
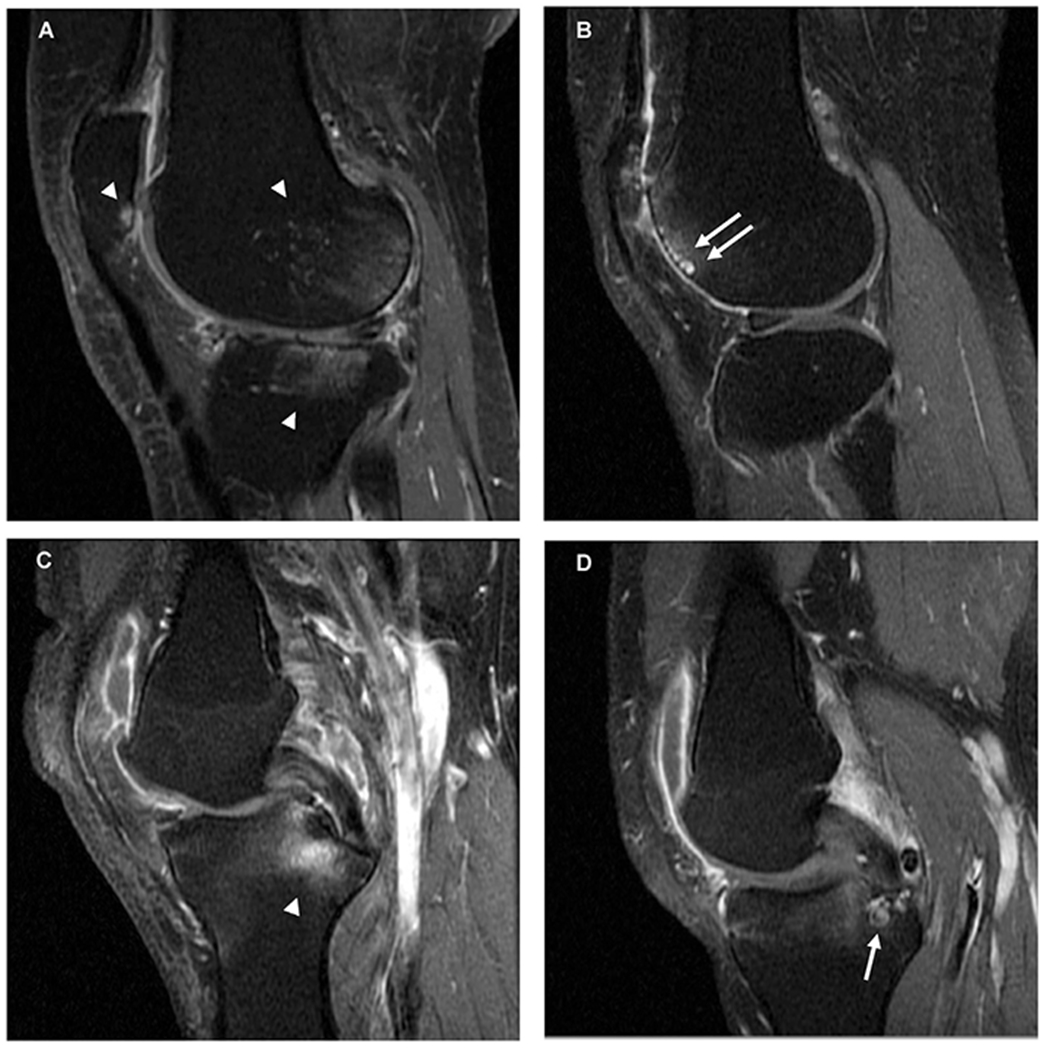
Bone marrow lesion (BML) types on sagittal T_1_-w fat suppressed contrast-enhanced MRI. (A) Subchondral BMLs at the posterior femoral condyle, posterior patella and lateral tibial plateau with ill-defined hyperintense (oedema-like) signal (white arrowheads). (B) Subchondral BML with cyst-like components (white arrows) in the anterior femoral condyle. (C) Ligament-based BML associated with the attachment of the posterior cruciate ligament (PCL) (odema-like signal extends into the subchondral bone) (white arrowhead). (D) Cyst-like component (white arrowhead) of a ligament-based BML in the tibia associated with the posterior cruciate ligament attachment.

Segmentation was performed using a manual approach with BML volume (mm^3^) segmented across the whole knee joint. BMLs, ill-defined hyperintense (oedema-like) regions on T_1_-w FS CE MRI, within the subchondral bone and those which appeared to be related to the attachment sites of the posterior cruciate ligament (PCL) or anterior cruciate ligament (ACL) were categorized into two subtypes (see Fig. 1). These subtypes included; i) those that did not include an internal region of well-defined, rounded hyper-intense signal (relative to oedema-like signal) on T_1_-w FS CE MRI and ii) those that did which we will refer to as a ‘BML with a cyst-like component’.

BMLs, both subchondral and ligament-based, were included for segmentation if present on 2 or more adjacent slices which is an accepted approach for the assessment of BMLs^18,19^. In order to classify a BML as subchondral in location, the ill-defined hyperintense signal which comprises the BML must have been adjacent to articular cartilage (or area of articular cartilage loss) on at least a single slice. BMLs within the patella were termed subchondral BMLs and were also included in assessment if meeting these criteria. BMLs were otherwise classed as ‘ligament-based’.

Within these two BML subtypes (subchondral or ligament-based), BMLs were further subdivided into those that contained a cyst-like component, or not. BMLs with a cyst-like component were defined if a single or multiple cyst-like component(s) were present on ≥2 adjacent slices, and the oedema-like signal appeared to be abutting or surrounding the cyst-like component(s). When calculating volumes for BMLs containing cyst-like component, the component’s volume was included.

The volume of the cyst-like component itself was segmented for subchondral BMLs with a cyst-like component only. Hyperintense signals within osteophytes that did not extend into the subchondral bone were excluded from volume measurement. Further, cyst-like components with no evidence of surrounding ill-defined hyperintense signal were also excluded from the volumetric measurement.

A total BML volume measurement for each region (subchondral/ligament-based) was calculated for each subject and visit by summing the volumes of both BML types (with/without a cyst-like component) for that given region. A total volume measurement was also calculated, comprising all BML volumes from both regions with or without a cyst-like component, and also including the cyst-like component volume (if present).

##### ii) Synovial Tissue Volume (STV)

STV was assessed across the whole knee joint using an approach previously reported^17^. In brief, STV was assessed using a semi-automated approach^20^ with STV and popliteal cysts segmented to the point where the gastrocnemius tendon was adjacent to the semi-membranosus tendon. In addition, where appropriate, images were manually truncated to control for differences in knee position within the field of view; this was performed on a per subject basis.

#### Statistical analysis

Descriptive statistics were used to summarise the characteristics of the study population with normally distributed variables summarised using means and standard deviations (SD) and non-normally distributed variables summarised using medians and inter-quartile ranges (IQR). Frequencies were described using percentages.

We used fixed-effects panel regression to assess the association between knee symptoms and the following volumetric parameters: i) total BML volume (sum of all BML types), ii) total subchondral BML volume, iii) total ligament-based BML volume, iv) volume of subchondral and ligament-based BMLs with, and v) without a cyst-like component, vi) volume of the cyst-like component itself from subchondral BMLs with a cyst-like component and vii) STV. Data across all available visits was included in analysis. The associations were reported as unstandardised B coefficients. The output of these models show that for a 1 unit increase in volume (mm^3^), a resulting change in WOMAC score is observed.

For each volumetric parameter, an additional dichotomous variable was created which indicated whether the participants had or did not have a BML volume for this parameter (coded 0 and 1, respectively). Including this variable allowed us to use the additional data from participants who did not have visible BMLs to be included in, and therefore increase the precision of, the estimate of within-person correlation in the outcome.

For each of the volumetric parameters, we ran a model with WOMAC pain (reported at all available visits) as the outcome variable. The explanatory variables were: the BML volume presence/absence indicator variable described above, the BML volume (across all available visits) of the parameter of interest, and visit (coded as 1 = baseline, 2 = year 1 and 3 = year 2). Visit was included as a continuous variable in the model, assuming the effect between symptoms and volume to be linear and constant over time; as consistent with previous studies^21^. To account for within patient correlations, patient identifier was set as the panel variable. The model was then repeated across all volumetric parameters, and also for the other WOMAC scores (function, stiffness and total WOMAC score) which were used as the outcome variable in turn instead of WOMAC pain. The presence/absence variable term would therefore indicate how the WOMAC outcome variables differed between those who did/did not have BMLs, and the linear coefficient for BML volume would indicate, in those that did have BML volumes, how the WOMAC outcome variables altered as BML volume increased. All statistical analysis was completed using Stata/IC version 14.0 (Stata Corp., College Station, Texas, USA) with a two-sided *P* values of 0.05 and less considered statistically significant.

## Results

### Subjects

50 subjects were included in the current analysis. The mean age of those included in the analysis was 63.3 years (SD 6.5) and 74% were female. The baseline characteristics of the 50 study participants is shown in Table I. While all 50 had at least one follow-up MRI and a baseline scan with images for BML volumes, follow-ups were not present at all time points in all 50. BML volumes and STVs of the index knee were missing for 6 subjects (12.0%) at year 1 and 19 subjects (38.0%) at year 2. Missingness was caused in one case (i.e., one study visit from one study subject) because of incorrect image acquisition of the primary sequence (sagittal T_1_-w FS CE). The remaining missingness was because scans had not been performed. Whilst inclusion to the primary trial specified that all participants must have KL grade 2–3, upon re-evaluation of the radiographs some participants were re-graded at KL1 and KL4. In our analysis, 8 participates were re-graded to KL1 and 4 were re-graded to KL4.

45 subjects had evidence of BMLs. Of these, all had subchondral BMLs (without a cyst-like component) whilst 19 subjects had a further ligament-based BML (with or without a cyst-like component) within the same index knee. Of those with subchondral BMLs, 17 subjects also had a further subchondral BML with a cyst-like component. Of those 19 subjects with ligament-based BMLs, 6 subjects had ligament-based BMLs with a cyst-like component; the remaining 13 did not.

### BML volume and symptoms

There was no statistically significant association between total BML volume and knee pain (*b* = 0.3 WOMAC units, 95% CI −0.3 to 0.9), function (*b* = 0.5, 95% CI −0.1 to 1.2), stiffness (*b* = 0.2, 95% CI −0.5 to 0.9) or total WOMAC score (*b* = 0.5, 95% CI −0.1 to 1.0); see Table II. There was no statistically significant association also between total subchondral BML volume and pain (*b* = 0.3, 95% CI −0.3 to 1.0), function (*b* = 0.5, 95% CI −0.2 to 1.2), stiffness (*b* = 0.2, 95% CI −0.6 to 1.0) or total score (*b* = 0.5, 95% CI −0.2 to 1.1). Lastly, there was no statistically significant association between total ligament-based BML volume and any of the WOMAC scores including pain (*b* = 1.9, 95% CI −1.6 to 5.3), function (*b* = 2.8, 95 CI −1.0 to 6.5), stiffness (*b* = 0.2, 95% CI −3.9 to 4.3) or total score (*b* = 2.4, 95% CI −1.0 to 5.8).

### Volume of BML subtype and symptoms

There was no statistically significant association between the volume of subchondral BMLs without a cyst-like component and pain (*b* = −0.1, 95% CI −0.8 to 0.7), function (*b* = 0.4, 95% CI −0.4 to 1.1), stiffness (*b* = 0.2, 95% CI −0.7 to 1.0) or total score (*b* = 0.3, 95% CI −0.5 to 1.0). Further, there was no statistically significant association between the volume of subchondral BMLs with a cyst-like component and pain (*b* = 0.8, 95% CI −0.5 to 2.1), function (*b* = 0.1, 95% CI −1.3 to 1.6), stiffness (*b* = −0.1, 95% CI −1.7 to 1.5) or total score (*b* = 0.3, 95% CI −1.0 to 1.5); see Table III. Of those with subchondral BMLs with a cyst-like component, the volume of the subchondral cyst-like component itself was statistically significantly associated with pain (*b* = 51.8, 95% CI 14.2 to 89.3) and total score (*b* = 40.2, 95% CI 2.1 to 78.3) though not with stiffness (*b* = 22.0, 95% CI −26.2 to 70.3) and function (*b* = 38.9, 95% CI −3.7 to 81.5); as shown in Table III. The reported coefficients for these subchondral cyst-like components appear large (the coefficient of 51.8, for example implies that a 1 cm^3^ increase in subchondral cyst-like volume reflects a corresponding change in WOMAC pain score of 51.8 points), however this reflects the smaller size of these cyst-like components, which are typically much less than 1 cm^3^ in volume. No statistically significant association was observed between symptoms and the volume of ligament-based BMLs with or without a cyst-like component; see Table IV.

### Synovial tissue volume and symptoms

There was a statistically significant association between STV and pain (*b* = 2.2 WOMAC units, 95% CI 0.6 to 3.7), function (*b* = 2.1, 95% CI 0.3 to 3.8) and total score (*b* = 2.0, 95% CI 0.5 to 3.5) though there was no statistically significant association with stiffness (*b* = 1.1, 95% CI −0.8 to 3.1); see Table V.

## Discussion

In this study in which CE MRI was performed, among patients with symptomatic knee OA there was no significant association between total subchondral BML volume or total ligament-based BML volume and knee symptoms. Further, there was no association between the volume of subchondral and ligament-based BMLs when stratified by the presence/absence of a cyst-like component. We did however, observe an association between the volume of the cyst-like component itself from subchondral BMLs with a cyst-like component and knee symptoms. The reported coefficients for these subchondral cyst-like components are large (for example, the coefficient for WOMAC pain score was 51.8 points (95% CI 14.2 to 89.3, *p* = 0.01). However, this is due to the size of these cyst-like components, which are typically much less than 1 cm^3^ in volume. This coefficient, rescaled to reflect a change of 1 mm^3^ is 0.05 points (95% CI 0.01 to 0.09, *P* = 0.01). As has been observed previously, STV was associated with pain and impaired function.

Data from two cross-sectional studies which examined the relationship between well-defined high signal regions on non-contrast enhanced MRI, assessed using a semi-quantitative approach, and pain in knee OA showed no statistically significant association though in both the risk was increased and in one study was of borderline statistical significance (*p* < 0.06)^12,13^. In our study, using quantitative assessment, there was no association between the volume of the subchondral BMLs with a cyst-like component and pain though the volume of the cyst-like component itself was associated with pain. The reason for the discordant findings is unclear though may, in part, be due to differences in the populations studied and also the method of assessment (quantitative vs semi-quantitative). Another possibility is the type of MRI sequence used; it is not known whether cyst-like regions have the same appearance on non-contrast and contrast enhanced scans. The mechanism by which cyst-like regions is linked with pain is unknown though given the histologic findings suggesting the presence of necrotic bone^7^ it is possible that these areas are associated with increased nociceptive stimuli. For instance, there is evidence from histological study to suggest that pain molecules including substance P can be found within cystic lesions^22^.

We did not observe an association between total subchondral BML volume and pain which is in contrast to the findings from large, observational studies^23,24^. To our knowledge, there have been no previous studies which have examined the association between the volume of ligament-based BMLs and knee symptoms on MRI.

Most data from observational and clinical studies support an association between STV and pain in symptomatic knee OA. Using data from the Boston Osteoarthritis of the Knee Study (BOKS), change in synovial thickening score was positively associated with an increase in knee VAS-pain on non-contrast enhanced MRI (*b* = 3.15 mm, 95% CI 1.04 to 5.26)^25^. Similarly, using data from the MOST study, synovitis score on CE MRI was positively associated with pain with those with extensive synovitis having up to a 5 fold increased risk (4.8, 95% CI 1.8 to 12.6) of mild pain (versus no pain) after adjusting for age, sex and BMI and, other structural outcomes of knee OA (BMLs/effusions)^26^. In a recent trial of intra-articular injection therapy (TASK study) in symptomatic knee OA, STV was associated with knee pain measured on both the KOOS questionnaire (*b* = −1.13, 95% CI −1.87 to −0.39), with a decrease in KOOS score indicative of a reduction in pain, and using a 10 cm VAS (*b* = 0.17, 95% CI 0.05 to 0.29)^27^. Wallace and colleagues showed using data from the UK VIDEO trial, that synovitis score, assessed semi-quantitatively^28^ on sagittal and axial T_1_-w FS CE MRI, was associated with an increased risk of knee pain (OR: 1.82, 95% CI 0.05 to 3.58, *P* = 0.04)^16^. Our findings, in which we used a quantitative approach to assessment, are consistent with these data.

There are a number of limitations which need to be considered in interpreting these findings. The number of subjects studied was relatively small reducing the likelihood of detecting significant biological associations between symptoms and structural parameters. This is compounded by the (necessary) complexity of the model used in our analysis. In our analysis we undertook a number of comparisons and it is possible that some of the observed findings may be due to type 1 error. The results, however, concerning the link between pain and STV is consistent with previous studies while the results relating to the link between symptoms and the volume of cyst-like components was significant across two of the four WOMAC domains reducing the likelihood of a statistical artefact. Further studies are, however, needed to confirm these findings. Another potential limitation was that we only included BMLs if appearing on ≥2 adjacent slices thereby potentially excluding small BMLs. Whilst this approach is consistent with the literature, this may have biased the results in relation to total BML volume towards the null, though the volumes involved would be expected to be small and probably unlikely to have influenced the overall outcome. A further limitation was that volumetric assessment of the cyst-like component was performed on CE MRI and therefore, the applicability of our findings in relation to studies using non-CE MRI is uncertain. Further, we did not perform segmentation of the cyst-like component from ligament-based BMLs.

## Summary and conclusions

Our data suggest that the volume of the cyst-like component itself from subchondral BMLs with cyst-like component on CE MRI are associated with pain in men and women with symptomatic knee OA. Future studies should include assessment of BMLs which includes information about the presence/absence of cyst-like components. In this study BML location did not influence the occurrence of knee symptoms.

## Figures and Tables

**Table I:** Subject characteristics at baseline of study population (*N* = 50)

Variable	Study Population (*N* = 50)
Age (years)	63.3 (6.5)
Females, n (%)	37 (74)
Index knee, n (% Right)	29 (58)
Body mass index (BMI) (kg/m^2^)	28.7 (4.9)
Worst Kellgren–Lawrence grade (medial/lateral) in index knee	
Grade 1, n (%)	8(16)
Grade 2, n (%)	22 (44)
Grade 3, n (%)	16 (32)
Grade 4, n (%)	4(8)
WOMAC* pain score	32.0 (17.7)
WOMAC stiffness score	48.0 (22.5)
WOMAC function score	35.9 (20.8)
WOMAC total score	36.1 (19.3)
Total Synovial tissue volume (mm^3^)	12,278.6 (6,185.0)
Total BML volume (mm^3^)	7,117.1 (9,347.3)
Total Subchondral BML volume (mm^3^)	6,434.5 (8,275.1)
Subchondral BMLs without a cyst-like component (mm^3^)	4,417.0 (5,955.2)
Subchondral BMLs with a cyst-like component (mm^3^)	2,017.5 (4,519.8)
Volume of the cyst-like component^†^ (mm^3^)	64.8 (254.9)
Total Ligament-based BML volume (mm^3^)	682.6 (1,813.4)
Ligament-based BML without a cyst-like component (mm^3^)	294.7 (1,096.9)
Ligament-based BMLs with a cyst-like component (mm^3^)	387.9 (1,522.6)

Results are shown as mean (SD) or frequencies (%).

Volumes reported here correspond to the mean, summed volumes of the given structure in those with the respective structure.

* Western Ontario and McMaster Universities Osteoarthritis Index (WOMAC): visual analogue scale (VAS) used to score pain, stiffness and total respectively from 0 to 100 units (0 = no pain/disability, 100 = high pain/disability).

† Volume of the cyst-like component itself from subchondral BMLs with cyst-like components.

**Table II:** Association between BML volume (total, subchondral & ligament-based) and symptoms

MRI parameter	Outcome			
	b (95% CI), *P* value			
	WOMAC^†^ pain score	WOMAC stiffness score	WOMAC function score	WOMAC total score
Total BML volume^‡,^* (*N* = 45)	0.3 (−0.3 to 0.9), 0.26	0.2 (−0.5 to 0.9), 0.63	0.5 (−0.1 to 1.2), 0.11	0.5 (−0.1 to 1.0), 0.13
Total subchondral BML volume^‡,^* (*N* = 45)	0.3 (−0.3 to 1.0), 0.31	0.2 (−0.6 to 1.0), 0.64	0.5 (−0.2 to 1.2), 0.15	0.5 (−0.2 to 1.1), 0.16
Total ligament-based BML volume^‡,^* (*N* = 19)	1.9 (−1.6 to 5.3), 0.29	0.2 (−3.9 to 4.3), 0.93	2.8 (−1.0 to 6.5), 0.15	2.4 (−1.0 to 5.8), 0.17

All results presented with 95% confidence intervals (CI).

*N*-values correspond to the number of participants included in each analyses.

* Volumes include cyst-like component volume.

† WOMAC index; range from 0 to 100 units with higher scores denoting worse pain and disability.

‡ Beta coefficients are scaled to reflect a unit change in WOMAC score (0–100) for a 1 cm^3^ increase in volume in the structural parameter.

**Table III:** Association between subchondral BML volume, with or without a cyst-like component, and symptoms

MRI parameter	Outcome			
	b (95% CI), *P* value			
	WOMAC^†^pain score	WOMAC stiffness score	WOMAC function score	WOMAC total score
Subchondral BMLs without a cyst-like component^‡^ (*N* = 45)	−0.1 (−0.8 to 0.7), 0.86	0.2 (−0.7 to 1.0), 0.71	0.4 (−0.4 to 1.1), 0.37	0.3 (−0.5 to 1.0), 0.48
Subchondral BMLs with a cyst-like component^‡,^* (*N* = 17)	0.8 (−0.5 to 2.1), 0.21	−0.1 (−1.7 to 1.5), 0.92	0.1 (−1.3 to 1.6), 0.85	0.3 (−1.0 to 1.5), 0.68)
Volume of the cyst-like component in subchondral BML^‡^ (*N* = 17)	51.8 (14.2 to 89.3), 0.01	22.0 (−26.2 to 70.3), 0.37	38.9 (−3.7 to 81.5), 0.07	40.2 (2.1 to 78.3), 0.04

All results presented with 95% confidence intervals (CI).

*N*-values correspond to the number of participants included in each analyses.

* Volumes include cyst-like component volume.

† WOMAC index; range from 0 to 100 units with higher scores denoting worse pain and disability.

‡ Beta coefficients are scaled to reflect a unit change in WOMAC score (0–100) for a 1 cm^3^ increase in volume in the structural parameter.

**Table IV:** Association between ligament-based BML volume, with or without a cyst-like component, and symptoms

MRI parameter	Outcome			
	b (95% CI), *P* value			
	WOMAC^†^ pain score	WOMAC stiffness score	WOMAC function score	WOMAC total score
Ligament-based BMLs without a cyst-like component^‡^ (*N* = 13)	2.6 (−2.2 to 7.4), 0.29	2.6 (−3.0 to 8.3), 0.36	3.0 (−2.2 to 8.3), 0.26	2.9 (−1.9 to 7.6), 0.23
Ligament-based BMLs with a cyst-like component^‡,^* (*N* = 6)	1.1 (−3.9 to 6.0), 0.67	−2.5 (−8.4 to 3.4), 0.4	2.5 (−2.9 to 7.9), 0.36	1.8 (−3.1 to 6.7), 0.47

All results presented with 95% confidence intervals (CI).

*N*-values correspond to the number of participants included in each analyses.

* Volumes include cyst-like component volume.

† WOMAC index; range from 0 to 100 units with higher scores denoting worse pain and disability.

‡ Beta coefficients are scaled to reflect a unit change in WOMAC score (0–100) for a 1 cm^3^ increase in volume in the structural parameter.

**Table V:** Association between synovial tissue volume (STV) and symptoms

Outcome	Synovial Tissue Volume (*N* = 50), b^†^ (95% CI), *P* value
WOMAC* pain	2.2 (0.6–3.7), 0.006
WOMAC stiffness	1.1 (−0.8 to 3.1), 0.24
WOMAC function	2.1 (0.3 to 3.8), 0.02
WOMAC total	2.0 (0.5–3.5), 0.01

All results presented with 95% confidence intervals (CI).

*N*-value corresponds to the number of participants included in each analyses.

* WOMAC index; range from 0 to 100 units with higher scores denoting worse pain and disability.

† Beta coefficients are scaled to reflect a unit change in WOMAC score (0–100) for a 1 cm^3^ increase in volume in the structural parameter.
